# Modeling and Targeting Neuroglial Interactions with Human Pluripotent Stem Cell Models

**DOI:** 10.3390/ijms23031684

**Published:** 2022-01-31

**Authors:** Julie Bigarreau, Nathalie Rouach, Anselme L. Perrier, Franck Mouthon, Mathieu Charvériat

**Affiliations:** 1 Theranexus, 69008 Lyon, France; julie.bigarreau@theranexus.com (J.B.); franck.mouthon@theranexus.com (F.M.); 2 Neuroglial Interactions in Cerebral Physiology and Pathologies, Center for Interdisciplinary Research in Biology, Collège de France, CNRS, INSERM, Labex Memolife, Université PSL, 75005 Paris, France; nathalie.rouach@college-de-france.fr; 3 Université Paris-Saclay, Commissariat à l’Energie Atomique et aux Energies Alternatives, CNRS, Laboratoire des Maladies Neurodégénératives: Mécanismes, Thérapies, Imagerie, 92265 Fontenay-aux-Roses, France; anselme.perrier@cea.fr; 4 Université Paris-Saclay, Commissariat à l’Energie Atomique et aux Energies Alternatives, Molecular Imaging Research Center, 92265 Fontenay-aux-Roses, France

**Keywords:** human pluripotent stem cells, neurons, astrocytes, microglia, neuroglial interactions, pathological modeling

## Abstract

Generation of relevant and robust models for neurological disorders is of main importance for both target identification and drug discovery. The non-cell autonomous effects of glial cells on neurons have been described in a broad range of neurodegenerative and neurodevelopmental disorders, pointing to neuroglial interactions as novel alternative targets for therapeutics development. Interestingly, the recent breakthrough discovery of human induced pluripotent stem cells (hiPSCs) has opened a new road for studying neurological and neurodevelopmental disorders “in a dish”. Here, we provide an overview of the generation and modeling of both neuronal and glial cells from human iPSCs and a brief synthesis of recent work investigating neuroglial interactions using hiPSCs in a pathophysiological context.

## 1. The Role of Glial Cells in the Brain

Although glial cells have long been considered only as supportive to neuron functions, the past two decades have witnessed a growing interest in these cells—namely, astrocytes, microglial cells, and oligodendrocytes, as their functions overlap with those historically ascribed to neurons [1]. Glial cells have indeed been involved in the development of numerous conditions of the central nervous system (CNS) [2].

Among glial cells, astrocytes are the most abundant glial cell type in the brain. Historically, their main role is to provide support to neurons and maintain brain homeostasis, and as such they are in charge of the clearance of ions and neurotransmitters, metabolic support, regulation of the blood–brain barrier, or modulation of neurogenesis [3,4,5]. Importantly, they integrate neuronal firing and synaptic transmission and thereby modulate neuronal activity within the so-called “tripartite synapse” [6]. More generally, they control synapse formation, maturation, and elimination, as well as neuronal functioning [5,7,8] and thereby participate to cognitive processes such as memory [9]. During pathological processes, astrocytes can play multiple roles, either neuroprotective or neurotoxic via for instance release of inflammatory cytokines, as demonstrated in epilepsy [10], Rett syndrome (RTT) [11], multiple sclerosis (MS) [12], or Alexander’s disease [13]. Modulation of their functions has also been described during treatment with CNS drugs [14,15,16,17,18].

The role of oligodendrocytes has been evaluated beyond its classical contribution to myelin formation on axons, and notably on how they support the long-term integrity of myelinated axons [19], the provision of neurotrophic factors and the regulation axon diameters, and the distribution of ion channels along those axons [20]. Oligodendrocytes are vulnerable to brain injury, as described in stroke [20]; in MS, dysfunction and apoptosis of oligodendrocytes lead to demyelination and neurodegeneration [21].

Furthermore, microglia, initially only considered as the immune cells of the brain, are now seen as crucial in the processes of neuronal patterning and synaptic wiring [22]. In their “resting state”, microglia act as immune surveyors and clear cellular debris in the brain; they secrete cytokines and express neurotransmitter receptors similar to the ones present in neurons [23]. As such, they are involved in synaptic pruning, i.e., the elimination of synapses during brain development [24,25]. When they are “activated”, microglial cells undergo morphological changes and move towards the sites of inflammation [26].

**Figure 1:**
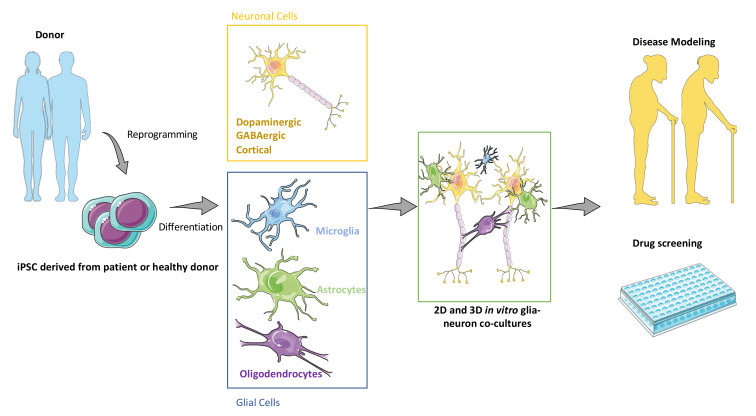
Overview of neuronal and non-neuronal cell production from human iPSCs.

Modeling neuroglial interactions at physiological and pathological levels is hence of paramount importance from both fundamental and translational perspectives in drug discovery [14,18,27,28,29,30]. This field largely benefited from the work of two Nobel Prize laureates, Pr. John B. Gurdon [31] and Pr. Shinya Yamanaka [32], through their discovery that mature cells can be reprogrammed to become pluripotent. This breakthrough led to the possibility to derivate patient-specific cells, typically fibroblasts, into pluripotent cells, called induced pluripotent stem cells (iPSCs) [33]. These stem cells can be further differentiated into active neurons [34], but also into astrocytes, oligodendrocytes, and microglia [27]. Hence, iPSCs represent a unique source of human brain cells, either obtained from healthy volunteers or patients with CNS disorders (Figure 1).

This review first focuses on the development and validation of protocols to produce neurons and glial cells from human iPSCs and how, when co-cultured, these models help better characterize neuroglial interactions in healthy and pathological conditions.

## 2. Generating Major CNS Cell Types Using iPSCs

To better understand neuroglial interactions, working on tissue and cells from humans as opposed to rodents is of paramount importance. Glia-to-neuron ratios indeed largely differ between these species (0.4 in rat [35] and 1.4 in human [36] in the cerebral cortex). Additionally, human glial cells and notably astrocytes, feature specific morphology, diversity, and functions compared with other species [37]. Additionally, human astrocyte-associated genes are less conserved than neuronal ones throughout evolution [38]. In addition, microglia from rodents and human show different aging processes [39], and oligodendrocytes from different species feature diverse maturation profile [40] and functions [41]. This section focuses on the generation of mature neuronal and glial cells specifically from human iPSCs.

### 2.1. Generation of Neuronal and Glial Derivatives from Human PSCs

Generation of neuronal or glial subtypes from human iPSCs has been achieved the past two decades largely via the sequential and timed application of extrinsic morphogen signals previously identified in vertebrate models of brain development, and additionally often successfully tested in vitro on mouse embryonic stem cells (mES).

#### 2.1.1. Modeling Human GABAergic with Medium Spiny Neurons

During human neural development, the most anterior part of the brain, the telencephalon, separates into two main regions: the pallium, dorsally, and the subpallium, ventrally. The first gives rise to the cerebral cortex and the hippocampus, the second to lateral, caudal, and medial ganglionic eminences (LGE, MGE, CGE) from which emerge notably the striatum and most cortical interneurons that migrate dorsally to the neocortex [42]. Key marker genes to characterize the different telencephalic progenitors are FOXG1, PAX6, GSX2, and NKX2.1. The vast majority (>90%) of striatal neurons are medium spiny neurons (MSN) projection neurons [43], with DARPP-32 as a canonical marker gene. Human MSNs are identified by co-expression of DARPP32, CITP2, FOXP1, ISL1, and GAD1. MSNs also broadly express calbindin [44]. Building on results of studies with mouse and human ES cells [45,46], the first protocol capable of generating MSN from hESCs was reported in 2008 [47]. This protocol was later optimized by the use of Dual SMAD inhibition to trigger more efficiently the neural induction [48] and by the dose refinements of key morphogens [49]. Alternatively, another study took advantage of the spontaneous “direct” neural induction protocol [50] to design another striatal differentiation protocol [51]. Moreover, SHH and WNT inhibitor-dependent protocols for the striatal differentiation of iPSCs were described by other groups [52,53]. In 2015, a completely novel strategy for the generation of MSNs from iPSCs was described, not based on the use of SHH-signaling but through activin A [54]. However, so far, the Wu et al. protocol remains as the one with the highest yield and fastest MSN differentiation of iPSCs [55].

#### 2.1.2. Cortical Neurons Generated from Human iPSC

The human cerebral cortex is organized into six-layers, each consisting of a mix of excitatory glutamatergic projection neurons, inhibitory GABAergic interneurons, and glial cells. Recapitulation of human cortical development, in a dish, using iPSCs as starting material, has been extensively explored in the past decades. As for striatal differentiation protocols for iPSCs, studies on mouse ESC intrinsic pathways of corticogenesis [56] were used to design the first protocols for the derivation of cortical neurons from iPSCs. These first protocols established that iPSC-derived telencephalic progenitor cells could generate functional pyramidal neurons of all six-layer identities [57,58].

The first cortical differentiation protocol for iPSCs was described by the FJ Livesey lab in 2012 [56] and showed that adherent human cortical progenitor cells can be produced from iPSCs via a neural induction step mediated by Dual SMAD inhibition. These cortical progenitors are identified by their expression of FOXG1, PAX6, OTX2, and BRN2. An alternative “adherent” cortical differentiation protocol was then described, based on prolonged treatment of iPSCs culture on laminin with noggin in absence of any other SMAD, WNT, or SHH inhibitors or RA [58]. More recently, several publications described an optimized version of adherent cortical differentiation protocols for iPSCs, with the use of cyclopamine [49] or DAPT [59]. The overall average cortical culture derived from iPSCs across labs yields a large majority of glutamatergic neurons (75–85%), a small fraction of GABAergic neurons (0–15%), and a limited number of astrocytes (5–10%).

Almost in parallel to the development of “adherent” cortical differentiation protocols for iPSCs, “cortical organoids” protocols were described. These protocols involve the differentiation of iPSCs into 3D free-floating aggregates (embryoid bodies) that become cortical organoids over time. The first true cortical organoids produced from iPSCs were described in 2013 [60]; since then, multiple refinements and optimization of cortical organoid protocols have been published [61].

Human iPSC-derived cortical neurons have been extensively used for disease modeling in classical 2D cultures [57,58], as 3D cortical organoids [61], or as neuronal networks reconstructed in microfluidic device [59]. The absence of sharp boundaries distinguishing cells from the six cortical layers in all those cultures and the absence of protocols to significantly enrich iPSC-derived cortical culture in neurons from specific layers still hamper finer modelling of human cortical function using iPSC derivatives.

#### 2.1.3. Producing and Characterizing Ventral Midbrain Dopaminergic Neurons

Dopaminergic neurons (DA neurons) are involved in the initiation and the control of motor functions, reward behavior, and cognition and their dysregulations and loss are hallmarks of Parkinson’s Disease (PD) and are associated with psychiatric disorders [62]. Therefore, intense efforts to generate mesencephalic dopaminergic neurons (mesDA) from human iPSCs have been gathered.

The identification of human ventral midbrain dopaminergic neurons often relies on the expression of tyrosine hydroxylase (TH) [63]. Nevertheless, TH is not specific to mesDA neurons [64]. Consequently, researchers have relied on the use of a selection of usual markers, including PITX3, LMX1A, FOXA2, CORIN, and OTX2 [65]. However, recent work showed that the number of dopaminergic progenitors expressing LMX1A, FOXA2, and CORIN does not correlate with the yield of dopaminergic neurons obtained after terminal differentiation in vivo or in vitro [66]. These authors established that the use of EN1, CNPY1, and BARHL1 in combination with the usual markers predicted more accurately mesDA yield [66]. Beyond the use of markers genes that define the commitment of progenitor cells to the mesDA lineage, the expression of DA biosynthesis enzyme and of the DA recapture protein DAT1 best characterize mature and functional mesDA neurons. Meanwhile, in vivo, mesDA display specific action potential patterns, and the detection of the robust electrophysiological signature of mesDA is still challenging in iPSC-derived cultures [67].

Initial protocols for deriving mesDA from iPSCs used poorly efficient neural induction methods relying on co-culture with stromal cells [46] and the use of SHH and FGF8 signals for the induction of ventral midbrain identity. The true breakthrough in the generation of mesDA neurons came from the description of the generation of floor plate cells from iPSCs [68], a protocol later applied for differentiation of iPSC into mesDA neurons [69]. In addition to the neuroectodermal induction step mediated by the inhibition of both SMAD main pathways, human mesDA neurons production requires the combined temporal and concentration-dependent activation and/or inhibition of transcription factors and morphogens coming from signaling centers [70]. Among all the protocols that describe the generation of ventral dopaminergic neurons (that are summarized in Table 1), few seemed to recapitulate dopaminergic functions, pointing out the need for new protocols [48,71,72,73].

### 2.2. Validated Protocols for Human Astrocytes Production

A chemical system for generating astrocytes from iPSCs was the first protocol described [74,75]. The general approach for this protocol includes three main phases: (i) the neural induction with the classical dual SMAD inhibition, (ii) the generation of astrocytic progenitors with the use of FGF2 and the epidermal growth factor (EGF), and (iii) a maturation step with the ciliary neurotrophic factor (CNTF). Despite the high yield of cells obtained, this protocol faces many hurdles. The total length of this protocol lasts for approximately 90 days and results in non-mature astrocytes that express GFAP and CD44 by week 12. Additionally, cells were considered as rather pure (with no contamination of microglia) and displayed glutamate receptor expression, calcium waves, and proofs of synaptogenesis [74,75].

Efforts for improving protocols for generating astrocytes have been multiple (Table 2). Despite a wide discrepancy of the growth factors used, the culture conditions (in suspension or adherent cells), and the duration of the protocols, they all share the induction of the neuroectoderm and the generation of neural progenitor cells (NPC) during the first 3 weeks. Recently, a group has screened the capacity of 42 NPC lines to generate astrocytes by testing nine different published protocols and three commercial media. Among the different screening conditions, most of them allow the generation of cells that are both positive for GFAP and S100B. The authors also reported that the astrocytes can display calcium wave and phagocytosis, as well as the release of interleukins upon stimulation [76]. In parallel, the adaptation of a previous protocol [77] led to an ameliorated protocol allowing the generation of mature and functional astrocytes that are easily produced for high-throughput screening of chemical compounds [78]. A long list of protocols has now emerged with the demonstration of the feasibility of differentiation of disease-associated iPSC lines for different CNS disorders.

### 2.3. Making Progress towards Human Microglia from iPSCs

Although multiple protocols for generating astrocytes have been reported, only a few studies have been focused on microglia derived from iPSCs (see Table 2). The first attempts to generate microglia from mouse were based on driving murine iPSCs towards the neuroectodermal lineage [79]. However, ontogenic studies highlighted that microglia arise from mesodermal primitive yolk sac progenitors rather than from neuroectodermal lineage [80]. Recently, several protocols have been reported for the successful generation of microglia derived from iPSCs [81,82,83]. Even if these protocols share many cytokines for driving the iPSCs towards the mesodermal/hematopoietic progenitor fate, they display multiple discrepancies in culture conditions, duration, yield, and purity. As an example, Muffat and colleagues generated microglia from iPSCs in 74 days, with 97% of cells that express key microglial proteins such as TMEM119, P2Y12, IBA1, and CD45 [81]. More recently, Abud and colleagues presented a method for a 65-day generation of mature microglia via an intermediate and freezable stage of hematopoietic progenitor cells [83].

Assessing the functionality of these cells, and therefore their maturity, is also crucial. Microglia produced from human iPSCs hence demonstrated key functional properties of the microglial cells, such as their response to inflammatory signaling, the ability of the cells to migrate, their phagocytic capabilities, and their ADP-dependent calcium response [81,82,83,84].

### 2.4. Human Oligodendrocytes: Still a Challenging Task

Multiple protocols for generating oligodendrocytes from iPSCs have been set-up by different groups (Table 2). Generating oligodendrocytes is both motivated by their potential use for cell replacement therapies for demyelinating disorders [85] and to model their pathophysiological role in cell culture [86]. One of the main methods to be reported was based on the use of murine iPSCs that can be differentiated into functional oligodendrocytes, as they were capable of myelin production both in vitro and in vivo [85]. Since then, multiple protocols have emerged and adapted to human iPSCs [87]. Most of the protocols validate the functionality of oligodendrocytes by evaluating the myelin production in vivo. However, little is known about the capacity of IPSCs-derived oligodendrocytes to produce myelin when co-cultured with neurons. In that sense, Ehrlich et al. transfected iPSCs with three transcription factors (Sox10, Olig2, and Nkx 6.2) and rapidly generated oligodendrocytes in 28 days [88]. These cells can produce myelin-like sheaths around axons when co-cultured with neurons derived from iPSCs and identified by myelin-basic protein expression. This is so far, and to our knowledge, the only validated method relying on transcription factors expression allowing the production of myelin in vitro.

## 3. Modeling Neuron-Glia Interactions with iPSCs

### 3.1. Physiological Role of Glial Cells

Glial cells are integral components of neural networks and play crucial roles in nearly all physiological processes via their structural and functional interactions with neurons. Glia cells are indeed tightly connected to neurons at the “quadripartite synapse” formed by the pre- and postsynaptic elements surrounded by astroglial and microglial processes [6,24,25] as well as at axons via the myelin sheath formed by oligodendrocytes [19]. These tight structural interactions are at the basis of a functional interplay underlying reciprocal cell activation and regulation. Glial cells and neurons share similar machineries for sensing and responding to activity. Glial cells indeed sense neuronal activity by using mostly ion channels, transporters, and receptors similar to the neuronal ones, and responding by transduction pathways, modulating in turn neighboring neurons by various mechanisms such as uptake or release of neuroactive factors, contact-mediated signaling, or plastic physical coverage [90,91,92]. These interactions thereby contribute not only to the regulation of extracellular homeostasis but also to active signaling. Up to now, most of our knowledge on glial cells and neuroglial interactions comes from investigations performed in in vitro cellular systems and animal models.

Evolutionarily, humans display an increased astrocyte/neuron ratio compared with lower vertebrates [37]. Nonetheless, the investigation of the complexity of human glial cells and their interactions with neurons still remains largely elusive. In fact, studies of human glial cells have been hindered by limited access to human brain cells, undefined molecular markers, and the difficulties in isolating and maintaining glial cells from adult human brains. The first studies revealing the complexity of human glia at the structural and functional levels were performed in tissues from postmortem brains or resected from patients with various pathologies [93,94].

### 3.2. Dissecting Neuroglial Interactions with iPSCs

In this context, hiPSCs with regenerative and multipotent features represent a very interesting model to gain insight into the nature of neuroglial interactions and the role of glial cells in the human brain. Until now, most studies investigating human glial cells derived them from hiPSCs of patients with various neurological disorders. These data thus advance our understanding of the role of glial cells in diseases. However, very little data are available about the nature of the neuroglial interactions and role of human glial cells in physiological conditions.

A few studies have started to unravel in normal conditions the properties and role of human glial cells, which display an extraordinary complexity and diversity compared with their rodent counterparts [93,94]. In vitro studies highlighted the role of human neuroglial interactions in the development of neuronal networks, as found in rodents. A recent study indeed showed that human astrocytes lineage cells contribute in vitro to the formation of neuronal networks derived from iPSCs by promoting synapse formation [95], as found for rodent astrocytes. Neuronal spontaneous network activity, assessed by synchronous calcium events, was indeed only found in cultures containing astrocyte lineage cells. Structural and functional investigations of neural networks depleted of glial cells indeed indicated that the absence of spontaneous network activity was associated with an immaturity of neurons, as suggested by their reduced dendritic arborization, density of excitatory synapses, AMPA receptor-mediated miniature excitatory postsynaptic currents, and neuronal excitability [95]. Remarkably, cholesterol, previously found to be released by astrocytes and to promote synapse maturation in rodents, was also reported to increase the density of excitatory synapses in these cultures derived from iPSCs and devoid of glia. Similar results were obtained with hiPSC-derived neural cells showing region specific phenotypes. For instance, retinal ganglion cells derived from iPSCs displayed an enhanced morphological and functional maturation during development in the presence of hiPSC-derived astrocytes [96]. Similarly, spinal cord hiPSC-derived astrocytes promoted the electrophysiological maturation of spinal cord hiPSC-derived motor neurons, and this occurred via changes in structural maturation and protein expression pattern of neurons [97].

Interestingly, analysis of hiPSC-derived neurons co-cultured with hiPSC-derived astrocytes also revealed that these latter favor the spiking of neurons, the formation and activity of their excitatory synapses and the networks they form by activating pathways involved in the function of AMPA and NMDA receptors, and the polarity of neurons and axon guidance, as revealed by single cell transcriptomics [98]. Functionally, it was also recently found that hiPSC-derived astrocytes temporally coordinate the spiking of iPSC-derived neurons [99]. These neurons indeed displayed more frequent and synchronized trains of spikes with dynamic patterns, indicating that astrocytes orchestrate the activity of neuronal circuits. The role of hiPSC-derived astrocytes in the overall structural and functional maturation of neurons is however likely to depend on their own maturation status, as a study reported the failure of hiPSC-derived astrocytes with an immature phenotype, as assessed with RNA-sequencing data, to enhance in vitro excitatory synaptic transmission of hiPSC-derived neurons [100]. Noteworthy, the astrocyte–neuron relation is reciprocal, as coculture of hiPSC-derived neurons also favors the maturation of hiPSC-derived astrocytes [97,101].

### 3.3. Towards New Tools to Assess Neuroglial Interactions In Vivo

However, culturing human glial cells in a dish is laborious and does not permit access to the endogenous neuroglial interactions occurring in a physiological environment. Remarkably, an ingenious and excellent model for studying the physiology of human glial cells and their interactions with neurons in a natural environment consists in transplanting human glial progenitor cells into mice, as performed in a pioneer study [102]. These progenitor cells were shown to migrate a long distance in the brain and mainly differentiate into astrocytes, which retained typical features of human astrocytes in the mice host brains, pointing to cell autonomous properties. These human astrocytes integrated functionally into the mice astroglial network, as they not only formed gap junctions with the host astrocytes, but also endfeet around mice blood vessels. The transplanted human astrocytes also showed higher input resistance and propagation speed of calcium signals and contacted more synapses than mice astrocytes. Functionally, these astrocytes were found to have a role in higher cognitive functions. The human-glia transplanted mice had indeed enhanced synaptic transmission, long-term potentiation, and learning capabilities. These data thus suggested early on a role for human astrocytes in the unique cognitive capabilities of humans. Since then, a few studies used the same approach to investigate the role of human astrocytes in different brain areas, such as the spinal cord [103] or somatosensory cortex [104]. In both regions, astrocytes derived from human neural progenitors also integrate well structurally and functionally into the host neural network, as assessed for instance by sensory-evoked calcium responses in engrafted astrocytes from the sensory cortex [104]. Remarkably, these engrafted astrocytes can also contribute to locomotion behavior [103] or sensory information processing [104]. Thus, human glial chimeric mice not only represent a powerful tool for fundamentally unravelling the species-specific characteristics of glia in regulating information processing and cognition but also point to the clinical potential of human glial graft for brain disorders [105].

## 4. Studying Pathophysiological Neuroglial Interactions Using Human Pluripotent Stem Cells

Neuroglial interactions are largely impaired in CNS disorders, notably through astrocyte morphological changes, microglial overactivation, or oligodendrocytes dysregulation [21]. Modeling the role of brain cells in CNS disorders using iPSCs, a concept known as “disease-in-a-dish” [106], has nourished new insights in the understanding of several pathogenesis processes. In this review, we focus on a few examples of studies on neuroglial interactions in these diseases (see Table 3).

### 4.1. Alzheimer’s Disease

Alzheimer’s disease (AD) is a neurodegenerative disorder characterized by the progressive and irreversible loss of cognitive functions. Brains from AD patients are distinguished by senile plaques essentially composed of amyloid beta protein (Aβ) and aggregates of hyperphosphorylated tau protein [107]. Stem cell-based forebrain neurons derived from patients were shown to recapitulate some AD phenotypes, such as Aβ accumulation, tau hyperphosphorylation, and reactive oxygen species increase [108,109]. Glial cells from AD patients also displayed pathological characteristics, such as reduced Aβ uptake or altered morphology for AD microglial cells [110], as recently reviewed [111]. In a recent study, astrocytes were also efficiently derived from iPSCs from AD patients carrying PSEN1 ΔE9 mutation. These cells showed higher Aβ production, altered mitochondrial function, and increased oxidative stress, as well as a reduction in lactate release, as demonstrated in AD patients. Similarly, astrocytes and neurons derived from iPSCs reprogrammed from sporadic AD patients confirmed this mitochondrial alteration [112]. Additionally, glutamate/glycine or GABA administration resulted in lower calcium-transient amplitudes in healthy neurons co-cultured with AD astrocytes as compared with healthy neurons co-cultured with healthy astrocytes. Thus, AD astrocytes exhibit a severe disease phenotype and deeply modulate neuronal activities, making them capable of contributing to AD pathogenesis [113]. The role of glial cells in AD has recently been modeled with astrocytes, neurons, and microglia derived from human hiPSCs, recapitulating several pathological features of this disorder [114].

### 4.2. Amyotrophic Lateral Sclerosis

Amyotrophic lateral sclerosis (ALS) is a rare neurodegenerative disorder characterized by the progressive loss of motor neurons in the brain and spinal cord [123]. Motor neurons derived from ALS patient iPSCs showed shorter neurites, increased TDP-43 aggregates [124], autophagy dysregulation [125], increased stress granules [126] and oxidative stress [127], and nucleocytoplasmic transports defects [128]. Regarding glial cells, ALS astrocytes exhibit aggregation, mis-localization of TDP-43, and decreased cell survival. Importantly, ALS astrocytes did not affect survival of cocultured neurons—either control or ALS neurons—providing new insights into ALS pathophysiological processes [129]. In parallel, patient-derived oligodendrocytes were shown to exacerbate motor neuron death via a SOD1-dependent mechanism, and interestingly via both soluble factors and cell-to-cell contact [86]. In addition, an ALS cellular model based on region-specific neurons and astrocytes co-cultured on microelectrode arrays has been developed to constitute a new drug testing platform in ALS [130].

### 4.3. Down Syndrome

Down’s syndrome (DS), caused by trisomy of human chromosome 21, is the most common genetic cause of intellectual disability [131]. Studies on human and transgenic mouse tissues have revealed impairments in neurogenesis and dendritic and synaptic morphology, as well as reduced brain volume and neuronal density. Regarding glial cells, iPSCs derived from DS patients allowed identification of the key role of astrocytes in the pathology, as they featured higher levels of reactive oxygen species, lower levels of synaptogenic molecules, and reduced neuronal viability and maturation [115].

### 4.4. Gaucher’s Disease

Gaucher’s disease (GD) type 2 is an acute neurological disorder characterized by an early-onset and severe neurological involvement of the brainstem, leading to death before the age of 2. GD is a lysosomal storage disorder caused by mutations in glucocerebrosidase 1 gene, resulting in the deficiency of the enzyme glucocerebrosidase (GCase) and the accumulation of the glycolipid substrates glucosylceramide and glucosphingosine [132]. Focusing on astrocytes derived from GD patients revealed the importance of these cells in the pathological processes, as they showed decrease in calcium signaling in response to ATP, reduction in GCase level and activity, and accumulated glucosylceramide [116]. Furthermore, excessive α-synuclein released from neurons was taken up by astrocytes and moved into lysosomes, as explored in PD [133].

### 4.5. Huntington’s Disease

Huntington’s disease (HD) is an autosomal dominant neurodegenerative disease characterized by movement disorders, behavioral and psychiatric symptoms, and cognitive decline over time. In neurons derived from patients, proteasome was impaired [134], mutant HTT was aggregated [135], and neuronal electrophysiological properties were altered [136]. Glia, and most possibly astrocytes and microglia, contribute to HD pathogenesis through transcriptional activation of pro-inflammatory genes, functional changes in glutamate and ion homeostasis, and finally to neuronal death [137]. Striatal neurons and astrocytes have been successfully derived from HD iPSCs; astrocytes exhibit impaired inward rectifying K^+^ currents, lengthened spontaneous calcium waves, and reduced cell membrane capacitance, as described in HD. More interestingly, astrocytes failed to demonstrate neuroprotective function after neuronal exposure to glutamate [117]. In addition, it was demonstrated that mitochondrial membrane potential and superoxide anion production were maintained in these cells, as demonstrated in isolated brain mitochondria [118]. Not all cellular dysfunctions mediated by HD mutations extend to glial cells. Indeed, nuclear pore deficits is identified in striatal neurons derived from patient iPSCs but not in astrocytes derived from the same cells [138].

### 4.6. Parkinson’s Disease

Parkinson’s disease is the second most prevalent neurodegenerative disease worldwide, affecting 10 million people worldwide; this neurological condition is notably characterized by the accumulation of toxic α-synuclein [139]. Patient-derived cultured dopaminergic neurons featured prominent α-synuclein, dendrite degeneration, and decreased tyrosine hydroxylase expression, as well as mitochondrial deficits, as experienced in the course of the disease [140,141]. Additionally, there is strong evidence that astrocytes accumulate α-synuclein during the course of the disease and that these proteins spread between astrocytes and neurons [133]. Modeling PD with patient-derived iPSCs, further differentiated into ventral midbrain dopaminergic neurons and astrocytes and co-cultured, confirmed these hypotheses. Control neurons were indeed less viable when co-cultured with PD astrocytes, and conversely the PD neuron phenotype was partially rescued by control astrocytes. Interestingly, further investigation pointed out the impairment of autophagy in PD astrocytes [119] and dysfunctional neuroglia interaction through alteration of extravesical biogenesis in astrocytes [142].

### 4.7. Schizophrenia

Schizophrenia (SZ) is a devastating mental disorder affecting 0.5% of the population worldwide [143]. Epidemiological evidence suggests that subtle perturbations in early neurodevelopment increase later susceptibility to SZ; neurons derived from SZ patients feature reduced neuronal connectivity, altered gene expression in glutamate pathway, impaired differentiation, and modification of mitochondrial functions, as recently reviewed [144]. Experimental studies also recently pointed out the major role played by glial cells in this disease, notably through abnormalities of differentiation competence of glial progenitor cells, leading to delayed and deficient maturation of astrocytes [145]. Very interestingly, co-cultures of neurons and microglial cells derived from SZ patients clearly demonstrated the contribution of glial cells to synaptic pruning in pathological situations [120].

### 4.8. Rett Syndrome

Rett syndrome is a rare genetic neurological disorder that affects brain development leading to mental and physical disabilities [146]. Cortical neurons derived from RTT patients display a lower number of synapses, reduced spine density, and smaller soma size compared with wild-type neurons [147]. Assessment of functionality of RTT neurons demonstrated reduced calcium signaling. Calcium homeostasis is also impaired in astrocytes derived from RTT patients [148].

### 4.9. Angelman Syndrome

Angelman syndrome (AS) is a neurodevelopmental genetic pathology characterized by intellectual disability, ataxia, and seizures [149]. Generation of neurons derived from AS-iPSCs has highlighted a defect in neuronal maturation and an impaired resting membrane potential leading to reduced neuronal activity [122]. Interestingly, hiPSC-derived cerebellar organoids have also provided new insights into the pathophysiology of AS, notably through the observation of a drastic reduction in GABAergic neuronal types in those models [150]. Angelman syndrome is linked with genetic alterations in the maternal UBE3A allele. Cell-type specific epigenetic silencing of the paternal UBE3A is hypothesized to result in neuronal dysfunctions associated with this syndrome [151]: this cell specificity further supports the use of different cell types in AS modeling and drug discovery.

## 5. Challenges and Perspectives

Drug discovery campaigns have been successfully performed using neurons derived from human iPSCs, notably in AD, on Aβ1-42-induced cellular toxicity [152], ALS with TDP-43 aggregation [153] and neuron survival [154], bipolar disorder on the modulation of Wnt/β-catenin signaling [155], or familial dysautonomia on the rescued expression of IKBKAP [156]. Furthermore, thousands of drugs have been tested on neurons from patients with Friedreich’s ataxia on the reactivation of the silenced Fmr1 gene [157], Niemann–Pick disease type C on lysosomal cholesterol accumulation [158], and PD on MEF2C activity [159]. The main goal of these screening studies was the constant search for drugs that might reverse the disease-associated phenotype [160]. Human neurons have also been envisioned to evaluate potential CNS side effects [161], and drug toxicity has been compared between iPSC-derived neurons and astrocytes [162]. Human astrocytes have primarily been screened to identify drugs active against oxidative stress [163]. Up to now, only a few studies have indeed focused on the use of mixed cultures with neurons and glial cells for drug discovery. As an example, a high-throughput co-culture assay was developed in ALS with the ability of astrocytes to support motor neuron survival as an outcome [164].

However, one must carefully address the potential limitations linked to the use of iPSCs, such as genetic variability and stability and epigenetic variations between different patients or healthy volunteers, or within the same subject [165,166]. On top of these difficulties, reproducibility, maturation, and differentiation should be carefully monitored, notably during large scale production [167]. In addition, the heterogeneity of glial cells, both spatially and temporally determined, constitutes a new challenge in the field, both under physiological and pathological conditions [168,169]. Exciting developments are on their way, notably with the foreseen use of iPSCs in personalized medicine, called “pharmaco-iPSCellomic”, as described in neurodegenerative [170] or neuropsychiatric disorders [171].

In addition, traditional 2D cultures are now being replaced by more relevant 3D cultures called organoids. These methods summarize the different steps of human cortical development, such as neurogenesis, gene expression profile, and regional and layer organization [172], and new protocols to produce myelinating oligodendrocytes, cortical neurons, and astrocytes have been recently released [173]. Organoids have successfully modeled CNS disorders, such as autosomal recessive primary microcephaly, brain infection by Zika virus, prenatal cocaine or nicotine exposure, or neonatal hypoxic stress [174], or recently traumatic brain injury [175] or ALS overlapping with frontotemporal dementia [176].

More generally, the use of iPSC-derived neuroglial cellular models are new powerful tools for improving our understanding of neuroglial interactions. They have indeed largely contributed to a better knowledge of the interplays between neurons, oligodendrocytes, microglial cells, and astrocytes, under both physiological and pathological conditions.

## Figures and Tables

**Table 1:** Differentiation of human iPSCs into dopaminergic neurons from different protocols.

Method of Differentiation	Key Markers	Functional Outcomes	Ref.
Noggin, SB, SHH, FGF8, BDNF, AA BDNF, GDNF, AA	TH, Tuj1	ND	[48]
Noggin, SB, SHH, GSK3i BDNF, GDNF, AA	MAP2, Synaptophysin, Lmx1a, FoxA2, Corin, TH	Electrophysiology (spontaneous action potential)	[71]
LDN, A83,01, Puromorphamine, GSK3i BDNF, GDNF, dbcAMP, AA	Nestin, Tuj-1, Corin, Nurr1, Pitx3, TH, FoxA2, Lmx1a, Nurr1, Otx2	Patch-clamp recording Dopamine release	[72]
Noggin, SB, SHH, CHIR, FGF8 BDNF, GDNF, AA, dbc-AMP	MAP2, Synaptophysin, Lmx1a, FoxA2, Corin, TH, Otx2	Not assessed *in vitro*	[73]

**Table 2:** Production of human glial cells from iPSCs.

Cell Type	Method of Differentiation	Key Markers	Functional Outcome	Ref.
**Astrocytes**	LDN, SB, EGF, FGF2, CNTF	GFAP, CD44, S100B, GLAST, NFIA, Aldh1L1	Glutamate uptake, Induction of synaptogenesis, Electrophysical recording	[75]
	Activin A, IGF1, Heregulin1b, FGF2	GFAP, S100b, CD44, NFIA, Vimentin	Glutamate Uptake, Inflammatory response, Calcium response, APOE secretion	[78]
**Microglia**	FGF2, BMP4, ActivinA, LiCl, VEGF, TPO, SCF, IL3, IL6, IL34, CSF, TGFB, CD200, CX3CL1	PU.1, TRM2, P2Y12, MERKT, CD11b, CD45	Synaptic pruning, Phagocytosis, ADP-dependent calcium imaging	[83]
	Commercial media IL34, TGFB, CD200, CX3CL1, CSF	PU.1, TRM2, P2Y12, MERKT, CD11b, CD45	Phagocytosis	[84]
**Oligodendrocytes**	RA, LDN, SB, SAG, PDGF, IGF1, HGF, NT3	Olig2, Nkx2.2, O4, MBP	ND	[89]
	SB, dorsomorphin, CHIR, purmorphamine, AA, SAG + lentiviral infection with 3 transcription factors (Sox10, Olig2 and Nkx 6.2)	Olig2, Nkx6.2, Sox10, O4, NG2, MBP	Myelin like sheaths production	[88]

**Table 3:** Examples of co-cultures of neurons and glial cells derived from iPSCs to model neurological disorders.

Indication	Cellular System	Main Outcome of the Study	Ref.
**AD**	Astrocytes derived from iPSCs from AD patients, co-culture with healthy neurons	Increased Aβ production, altered mitochondrial metabolism, and reduced lactate secretion in mutant astrocytes Alteration of calcium signaling in healthy neurons by mutant astrocytes	[113]
**ALS**	Motor neurons and oligodendrocytes derived from iPSCs from ALS patients	Increase in motor neuron death by ALS oligodendrocytes	[86]
**DS**	Neurons and astrocytes derived from iPSCs from DS patients	Abnormal morphology of neurite outgrowth Reduction in neuronal differentiation and survival when exposed to DS astrocytes	[115]
**GD**	Dopaminergic neurons and astrocytes derived from iPSCs from GD type 2 patients	Low GCase activity and accumulation of glucosylceramide in GD astrocytes Excessive α-synuclein from neurons is taken up by astrocytes and moved into lysosomes	[116]
**HD**	Striatal neurons and astrocytes derived from iPSCs from HD patients	HD astrocytes in co-culture provided reduced support for the maturation of iPSC-derived neurons HD neurons exposed to chronic glutamate stimulation are not protected by HD astrocytes	[117]
**HD**	Striatal neurons and astrocytes derived from iPSCs from HD patients	mHTT at early stages of HD pathology does not deteriorate mitochondrial functions	[118]
**PD**	Ventral midbrain dopaminergic neurons and astrocytes derived from iPSCs from familial mutant LRRK2 PD patients	Control astrocytes partially prevented the appearance of disease-related phenotypes in PD neurons Control neurons displayed morphological signs of neurodegeneration and abnormal, astrocyte-derived synuclein accumulation	[119]
**SZ**	Cortical neurons and microglia derived from iPSCs from SZ patients	Microglia increased synaptic elimination	[120]
**RTT**	Astrocytes and cortical neurons derived from RTT-iPSC	Altered calcium signaling in both neurons and astrocytes	[121]
**AS**	Neurons derived from AS-iPSC	Reduced calcium signaling, altered resting membrane potential	[122]
